# Supplementary material for: A Modified Protocol of Diethylnitrosamine Administration in Mice to Model Hepatocellular Carcinoma
Source: Int J Mol Sci. 2020 Jul 30;21(15):5461. doi: 10.3390/ijms21155461 (PMC7432842; doi:10.3390/ijms21155461)
Supplement: Supplementary file 1 [file ijms-21-05461-s001.pdf]

International Journal of *Molecular Sciences*

MDPI

Supplementary Figures

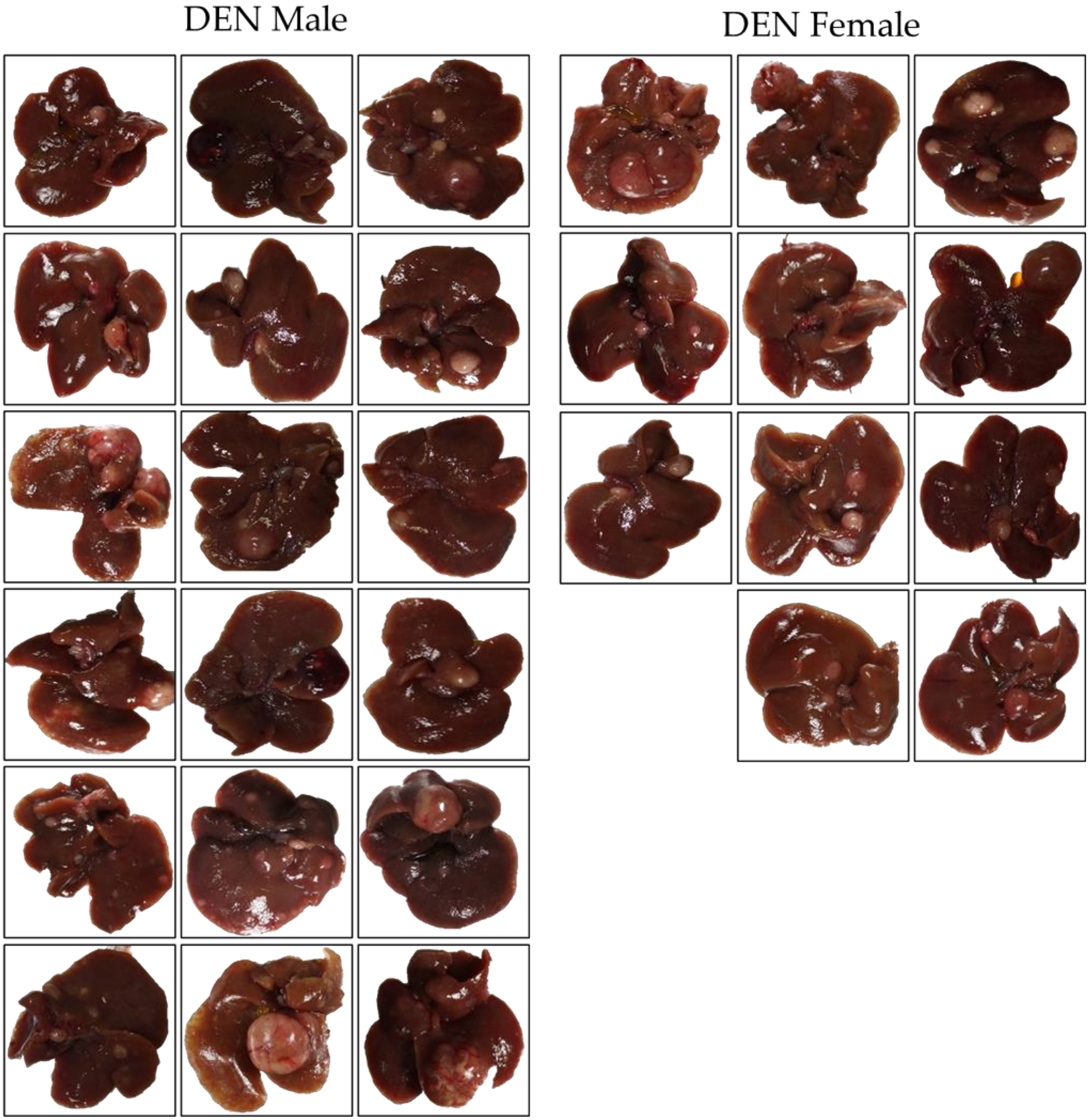

Figure S1. Macroscopic view of DEN-treated male and Female. Both male and female group shows a visible tumor in both male and female group.

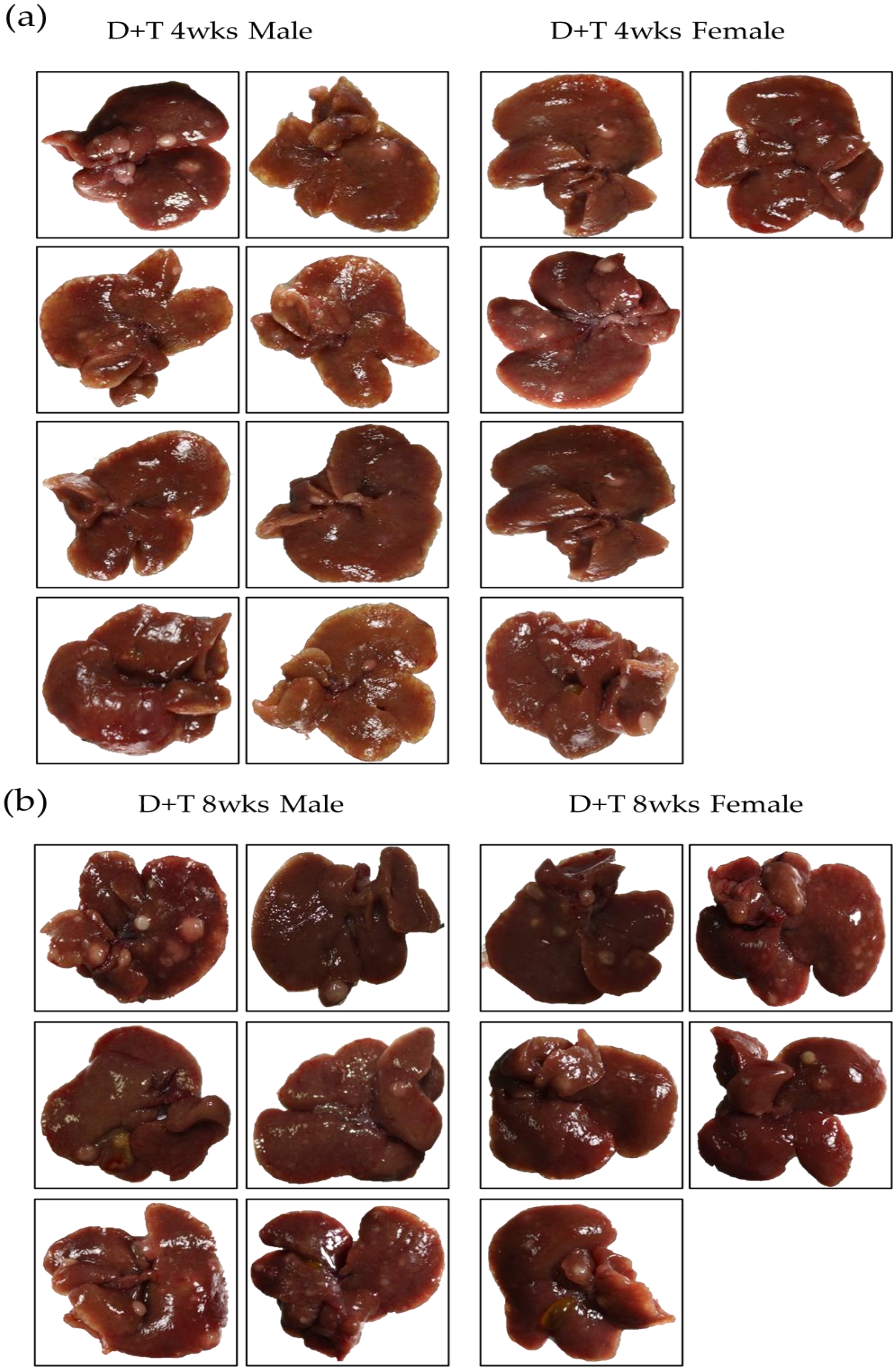
(a)
D+T 4wks Male
D+T 4wks Female
(b)
D+T 8wks Male
D+T 8wks Female

Figure 2. Macroscopic view of DEN+TAA treated groups. D+T4wks group (b) D+T8wks group both male and female groups shows a visible tumor as well as cirrhosis development in both 4wk and 8wk groups and male and female groups.

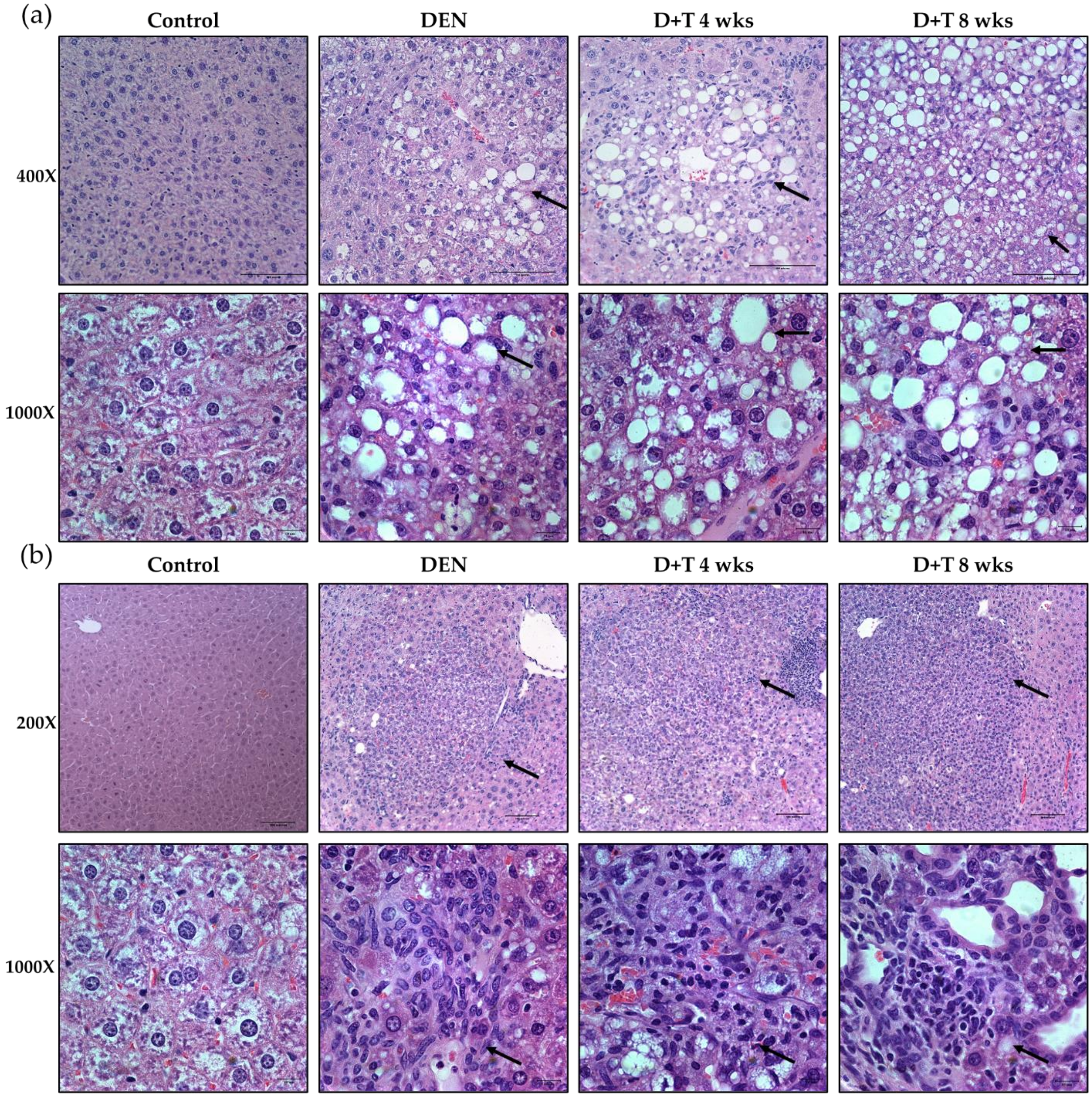

Figure S3. The cellular arrangement of HCC that developed in DEN, DEN+TAA 4 weeks, or 8 weeks group (a) some non-tumor area also showing numerous huge pathological vacuoles in hepatocytes suggesting those lipid droplets (indicated with black arrows) accumulated in the liver and that these animals developed hepatic steatosis (400X, & 1000X). (b) Presence of fibrosis showing basophilic foci with crowded nuclei (indicated with black arrows) in the H&E stained section (200X & 1000X).

Table S1. Tumor number in all treated groups according to gender.

Group	SEX	Mice # with tumor /total # of mice	Tumor NO.	Tumor NO./animal
Control	Male	0/7	0	0
	Female	0/6	0	0
DEN	Male	18/18	141	7.8±1.83##
	Female	11/11	63	5.73±1.49
DEN+TAA 4weeks	Male	8/8	67	8±1.11*,ns
	Female	5/5	39	8±1.32*
DEN+TAA 8weeks	Male	6/6	77	13±2.47**,#
	Female	5/5	49	10±0.74**

** Significantly different from the DEN group (p<0.01), * Significantly different from the DEN group (p<0.05),##Significantly different from the female group (p<0.01),#Significantly different from the Female group (p<0.05), ns (not significant).

Table S2. Time to develop the DEN model in previous studies.

Mice age		Dose	Treatment Time	Effect	References
15days old IP		25mg/kg	32 weeks	HCC in male	[1]
15days old IP		25mg/kg	36 weeks	10% HCC<br>100% adenoma	[2]
14 days of age IP		10 mg/kg	24 weeks<br>36 weeks	47% at 36week	[3]
15days old IP		5mg/kg	48 weeks	50 % HCC in wild	[4]
15days old IP		15mg/kg	36 weeks	At 36-week HCC	[5]
15days old IP		5ug/g +PB 0.5% in drinking water	32 weeks	40% HCC	[6]
4-week-old mice		100mg/kg +PB	32 week	55% HCC in mice	[7]
7-week-old mice		100, 50mg/Kg +CCL_4 5ml,8ml	8,12,16,20 weeks	20 visible nodules, HCC	[8]

Supplementary Reference

1. Naugler, W.E.; Sakurai, T.; Kim, S.; Maeda, S.; Kim, K.; Elsharkawy, A.M.; Karin, M. Gender disparity in liver cancer due to sex differences in MyD88-dependent IL-6 production. *Science* **2007**, *317*, 121-124, doi:10.1126/science.1140485.
2. Fan, Y.; Boivin, G.P.; Knudsen, E.S.; Nebert, D.W.; Xia, Y.; Puga, A. The aryl hydrocarbon receptor functions as a tumor suppressor of liver carcinogenesis. *Cancer Res.* **2010**, *70*, 212-220, doi:10.1158/0008-5472.Can-09-3090.
3. Kang, J.S.; Wanibuchi, H.; Morimura, K.; Wongpoomchai, R.; Chusiri, Y.; Gonzalez, F.J.; Fukushima, S. Role of CYP2E1 in thioacetamide-induced mouse hepatotoxicity. *Toxicol. Appl. Pharmacol.* **2008**, *228*, 295-300, doi:10.1016/j.taap.2007.11.010.
4. Riehle, K.J.; Campbell, J.S.; McMahan, R.S.; Johnson, M.M.; Beyer, R.P.; Bammler, T.K.; Fausto, N. Regulation of liver regeneration and hepatocarcinogenesis by suppressor of cytokine signaling 3. *J. Exp. Med.* **2008**, *205*, 91-103, doi:10.1084/jem.20070820.
5. Shang, N.; Bank, T.; Ding, X.; Breslin, P.; Li, J.; Shi, B.; Qiu, W. Caspase-3 suppresses diethylnitrosamine-induced hepatocyte death, compensatory proliferation and hepatocarcinogenesis through inhibiting p38 activation. *Cell Death Dis.* **2018**, *9*, 558, doi:10.1038/s41419-018-0617-7.
6. Awuah, P.K.; Rhieu, B.H.; Singh, S.; Misse, A.; Monga, S.P. β-Catenin loss in hepatocytes promotes hepatocellular cancer after diethylnitrosamine and phenobarbital administration to mice. *PLoS One* **2012**, *7*, e39771.
7. Maeda, S.; Kamata, H.; Luo, J.L.; Leffert, H.; Karin, M. IKKbeta couples hepatocyte death to cytokine-driven compensatory proliferation that promotes chemical hepatocarcinogenesis. *Cell* **2005**, *121*, 977-990, doi:10.1016/j.cell.2005.04.014.
8. Xin, B.; Cui, Y.; Wang, Y.; Wang, L.; Yin, J.; Zhang, L.; Pang, H.; Zhang, H.; Wang, R.A. Combined use of alcohol in conventional chemical-induced mouse liver cancer model improves the simulation of clinical characteristics of human hepatocellular carcinoma. *Oncol. Lett.* **2017**, *14*, 4722-4728, doi:10.3892/ol.2017.6800.
